# Simulating the impact of varying vegetation on West African monsoon surface fluxes using a regional convection‐permitting model

**DOI:** 10.1002/pei3.10107

**Published:** 2023-04-27

**Authors:** Adama Bamba, Kouakou Kouadio, N’Datchoh E. Toure, Lawrence Jackson, John Marsham, Alex Roberts, Masaru Yoshioka, Sandrine Anquetin, Arona Diedhiou

**Affiliations:** ^1^ Laboratoire des Sciences de la Matière, Environnement et Energie Solaire Université Félix Houphouët Boigny Abidjan Côte d'Ivoire; ^2^ Centre d'Excellence Africain en Changement Climatique, Biodiversité et Agriculture Durable (CEA‐CCBAD) Université Félix Houphouët Boigny Abidjan Côte d'Ivoire; ^3^ Laboratoire Mixte International sur le Nexus Climat‐Eau‐Energie‐Agriculture Université Félix Houphouët Boigny Abidjan Côte d'Ivoire; ^4^ School of Earth and Environment, Institute for Climate and Atmospheric Science University of Leeds Leeds UK; ^5^ Université Grenoble Alpes, IRD, CNRS, Grenoble‐INP, IGE Grenoble France

**Keywords:** convection‐permitting, evapotranspiration, heat fluxes, precipitation, seasonal vegetation

## Abstract

This study assessed the sensitivity of the West African climate to varying vegetation fractions. The assessment of a such relationship is critical in understanding the interactions between land surface and atmosphere. Two sets of convection‐permitting simulations from the UK Met Office Unified Model at 12 km horizontal resolution covering the monsoon period May–September (MJJAS) were used, one with fixed vegetation fraction (MF‐V) and the other with time‐varying vegetation fraction (MV‐V). Vegetation fractions are based on MODIS retrievals between May and September. We focused on three climatic zones over West Africa: Guinea Coast, Sudanian Sahel, and the Sahel while investigating heat fluxes, temperature, and evapotranspiration. Results reveal that latent heat fluxes are the most strongly affected by vegetation fraction over the Sahelian and Sudanian regions while sensible heat fluxes are more impacted over the Guinea Coast and Sudanian Sahel. Also, in MV‐V simulation there is an increase in evapotranspiration mainly over the Sahel and some specific areas in Guinea Coast from June to September. Moreover, it is noticed that high near‐surface temperature is associated with a weak vegetation fraction, especially during May and June. Finally, varying vegetation seems to improve the simulation of surface energy fluxes and in turn impact on climate parameters. This suggests that climate modelers should prioritize the use of varying vegetation options to improve the representation of the West African climate system.

## Graphical abstract

Comparison between the use of varying vegetation fraction and fix vegetation fraction in regional climate models over West Africa: Case study of the Unified Model with focus on three different climatic zones.

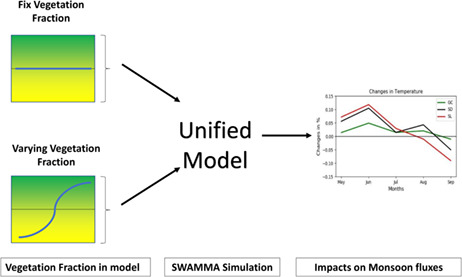

## 1 INTRODUCTION

West Africa is a region of diverse vegetation varying from tropical forests bordering the Gulf of Guinea coast in the south to the Sahara desert in the north with savannah and grassland of the Sahel in between. The interactions between the land surface and atmosphere strongly influence the variability in climate and land surface processes (Weiss et al., 2004). The land surface can influence evapotranspiration, soil moisture content, radiation flux partitioning, and aerodynamic roughness. These factors can in turn modify the weather and climate by interacting with atmospheric processes such as mesoscale circulation, initiation and development of convection, cloud formation, and subsequent precipitation (Weiss et al., 2004).

In turn, the climate exerts a dominant control on the spatial distribution of the major vegetation types from local to global scales through variation in rainfall and temperature. However, the socioeconomic activities across West Africa are heavily dependent on rainfall‐fed agriculture, and the population of the region is projected to have a strong increase during the twenty‐first century. Thus, according to the United Nations, Department of Economic and Social Affairs, Population Division (2019), sub‐Saharan Africa will account for most of the growth of the world's population over the coming decades. As discussed by Strengers et al. (2010), the land‐use and land‐cover change (LULCC) have been shown to be one of the most important drivers of changes in land surface properties in the past, and they are likely to trigger further changes in the future. Climate models have recently been used to investigate various processes such as land–atmospheric interactions (Bamba et al., 2019), climate dynamics (Kouadio et al., 2015), and aerosols radiative impacts (N'Datchoh et al., 2018). Furthermore, these climate models have been useful tools to understand the climate and its interaction. Thus, Walters et al. (2019) argue that the exchange of fluxes between the land surface and the atmosphere is an important mechanism for heating and moistening the atmospheric boundary layer. The different processes at the land–atmosphere interface are hardly reproduced by regional climate models (RCMs). According to Betts et al. (1997) and Bounoua et al. (2002) modeling, studies on global scales show that vegetated land interacts with the atmosphere to produce significant effects on regional climate. Also, Lu et al. (2001) find that the variability in vegetation phenology (timing of biological events) influences the regional climate through changes in surface moisture and energy balances.

In general, climate models are mainly limited by the use of prescribed annual vegetation which does not allow a realistic interaction between vegetated surfaces and atmospheric processes. For instance, the annual or biannual rains in Africa regularly transform the transitional regions between the desert and vegetated land from almost bare‐soil pre‐monsoon to lush green vegetation post‐monsoon (Mougin et al., 2014). In common with many similar models, these variations are not generally represented in the UK Met Office Unified Model (UM), the climate model used in Future Climate For Africa (FCFA) and the focus of this study, resulting in large systematic biases in its surface fluxes (heat, moisture, momentum, and dust), and providing largely unknown likely important errors. The UM normally uses fixed vegetation fractions that cannot capture the abovementioned changes (Best et al., 2011). This is an important assumption that likely weakens the role of vegetation in the physical processes of land–atmosphere interactions as the vegetation seasonality is generally not or only weakly taken into account. This can increase biases between observations and simulations, thus affecting the ability of a model setup to perform weather or climate prediction. Given the key role of the land surface and dust, the FCFA Improving Model Processes for African cLimAte (IMPALA) project includes improvements to dust and the land surface in the UM but did not plan to address this model deficiency. Since IMPALA was conceived, the UK's National Environment Research Council (NERC), Saharan West African Monsoon Multi‐Scale Analysis project (SWAMMA) has demonstrated that this gap provides a fundamental error in dust modeling.

Also, efforts within IMPALA to improve the dust‐generating winds via a haboob parametrization will not lead to the expected improvement unless it is addressed (Roberts et al., 2018). The land surface plays a key role in the West African monsoon, and so the seasonal variation in moisture fluxes introduced by seasonally varying vegetation fractions would also be expected to substantially affect regional climate (Nogherotto et al., 2013; Steiner et al., 2009). Yet, most climate models typically assume a fixed annual vegetation fraction. This VegFlux project, therefore, addresses a key gap in FCFA, which, if not addressed, will limit model developments being made in IMPALA. It will therefore facilitate a better fundamental understanding of land–atmosphere interaction, a better understanding of limits to current projections and allow improvement of future projections. In light of all above, the present study assesses the impact of the vegetation seasonal variation in surface features such as surface winds and temperature, evaporative fraction, and energy at the continental scale in the UM so we investigate the benefits of introducing seasonally varying vegetation cover in climate models. This is realized through UM simulations broadly following the model setup used in the SWAMMA (Section 2.2) and the Seasonally varying vegetation impacts on surface fluxes (VegFlux) project simulations In Section 2, we describe the model setup and the experiments performed as well as the observations used to validate the model. Results on the variations in temperature, latent and sensible fluxes, and evapotranspiration fraction are presented and discussed in Section 3. The conclusion is given in Section 4.

## 2 METHODOLOGY

### 2.1 Model description

The UM has been widely used and improved throughout studies and research programs (Marsham et al., 2016, 2013). Detailed descriptions of the UM version 8.2 used for the SWAMMA project are provided in several research papers (Kealy et al., 2017; Roberts et al., 2018), and a summary is given below (Walters et al., 2019). The model uses a mass flux convection scheme with various extensions to include down draughts and convective momentum transport (CMT). The aerosol species representation and their interaction with the atmospheric parametrizations are, however, part of the Global Atmosphere component. The exchange of fluxes between the land surface and the atmosphere is an important mechanism for heating and moistening the atmospheric boundary layer, as well as generating drag on atmospheric winds. The Global Land configuration uses a community land surface model, the Joint UK Land Environment Simulator (JULES; Walters et al., 2019) to simulate processes at the land surface and in the subsurface soil. JULES also uses a canopy radiation scheme to represent the penetration of light within the vegetation canopy and its subsequent impact on photosynthesis. Soil processes are represented using a four‐layer scheme for the heat and water fluxes with hydraulic relationships. Sub‐grid‐scale heterogeneity of soil moisture is represented using the large‐scale hydrology approach. A tile approach is used to represent sub‐grid‐scale heterogeneity, with the surface of each land point subdivided into five types of vegetation (broadleaf trees, needle‐leaved trees, temperate C3 grass, tropical C4 grass, and shrubs) and four nonvegetated surface types (urban areas, inland water, bare soil, and land ice).

### 2.2 Experiment design

Vegflux simulations are identical to the 12 km grid‐spaced convection‐permitting SWAMMA simulations apart from changes to the representation of vegetation type and fraction (described below). The simulations are run at a horizontal grid spacing of 12 km using convection‐permitting (Klein et al., 2021) setups during the summer of 2011 (1 May–30 September 2011), period which we refer to as MJJAS hereafter. Studies have shown this period as the West African monsoon (WAM) setting period characterized by more rainfall over the region (Bamba et al., 2019) The simulation configurations use a fully interactive mineral dust scheme though the Moderate Resolution Imaging Spectroradiometer (MODIS) and the EUMETSAT Spinning Enhanced Visual and Infrared Imager (SEVIRI), and the current work focused on land surface fraction types as described. The simulations do not include the radiative feedback from dust but take into account the climatological aerosol concentration and their radiative effects. The simulations encompass the entire West African domain (approximately 0–35° N and 23° W–35° E). For the two simulations, MODIS AOD and SEVERI AERUS‐GEO (Aerosol and surface albEdo Retrieval Using a directional Splitting method‐application to GEOstationary data) AOD were used and separately; the first one was performed using a fixed vegetation fraction referred to hereafter as MF‐V. For this simulation, the MODIS Leaf Area Index (LAI) was used to represent the vegetation fraction, which was averaged over May–September period. The second simulation was performed using a time‐varying LAI from MODIS which we refer hereafter as MV‐V. The daily varying LAI data cover the period from May 1 to September 30. A full description of the SWAMMA simulations is provided by Roberts et al. (2018). Thus, VegFlux is using the same configuration such as SWAMMA except the vegetation fraction treatment, which is invariant in SWAMMA and varies in VegFlux.

### 2.3 Data

SWAMMA model outputs have been validated using: satellite data from MODerate resolution Imaging Spectroradiometer (MODIS) and the Spinning Enhanced Visible and InfraRed Imager (SEVIRI) as well as station data of near‐surface wind observations from the Fennec field campaign (Roberts et al., 2018) and from the Analyse Multidisciplinaire de la Mousson Africaine—Couplage de l'Atmosphère Tropicale et du Cycle Hydrologique (AMMA‐CATCH) observatory in West Africa (Lebel et al., 2011). The validation processes are described in Roberts et al. (2018). Given that the VegFlux simulations are identical in every way to the SWAMMA simulations apart from the source and variability of vegetation type and cover, it was not deemed necessary to repeat the work of Roberts et al. (2018) (especially as the VegFlux simulation MF‐V has similarly invariant vegetation as used in the SWAMMA simulations). So, it is expected that differences would be very small, especially between the MF‐V and the SWAMMA simulations.

### 2.4 Methods

**FIGURE 1:**
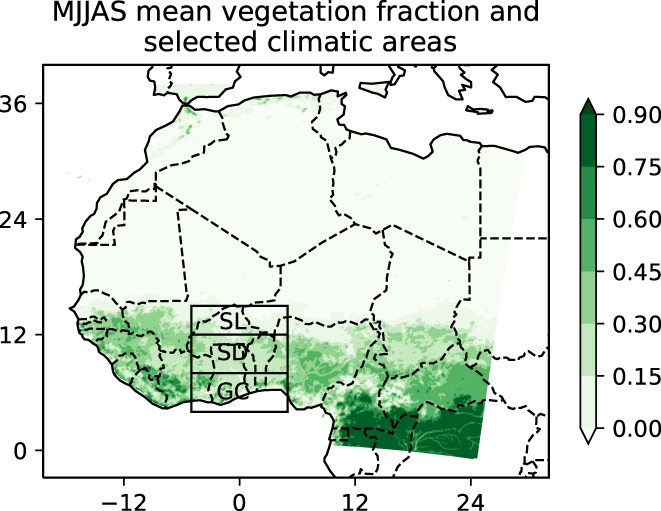
West Africa map showing the study area with the three selected climatic zones: Guinea Coast (GC), Sudanian zone (SD), and Sahel zone (SL). Vegetation fraction from MODIS observations is shown on a scale from 0 to 1.

Three subregions have been used in this work to account for the latitudinal variation of the regional climate, due to the annual migration of the Intertropical Convergence Zone (ITCZ), from tropical to semiarid climate zones across West Africa. The Sahel (SL) is located between 12° N and 15° N; the Sudanian zone (SD) is located between 8° N and 12° N; and, the Guinea coast zone (GC) is between 4° N and 8° N. All these subregions are located within 5° W–5° E (Figure 1).

The sensitivities of the UM are tested, from May to September, for changes in vegetation cover (fixed vegetation fraction vs. time‐varying vegetation fraction) on the simulation of the features of the West African climate, especially the surface fluxes monthly and diurnal precipitation, 1.5 m temperature, evaporation fraction (EF), sensible heat (SH) flux, and latent heat (LH) flux using mean bias (in %) and correlation coefficient pattern over the three subregions between the two different experiments. (SL, SD, and GC).

The sensitivity of the UM for the changes in vegetation cover on EF is also assessed. EF is one of the most widely used methods to estimate the daily evapotranspiration (ET; Nutini et al., 2014). It is defined as the ratio between LH and the total heat leaving the Earth's surface. Daily ET is estimated as the product of the daily available energy estimated from SEVIRI/MSG data and the instantaneous evaporative fraction (ET_frac_) estimated from Terra MODIS data. Further detail on ET_frac_ estimation can be found in Sun et al. (2012).

The ET_frac_ is computed based on Equation (1)

(1) $${\mathrm{ET}}_{\text{frac}}=\frac{\mathrm{LH}}{\mathrm{SH}+\mathrm{LH}}.$$

The significance of the changes induced by the variation of the vegetation fraction is evaluated through the comparison between the monthly mean of MV‐V and MV‐F (two independent samples). A *t*‐test with a 10% significance level is applied to MV‐V and M‐VF simulations in which the sample sizes are 5 months.

## 3 RESULTS AND DISCUSSIONS

### 3.1 Vegetation fraction

**FIGURE 2:**
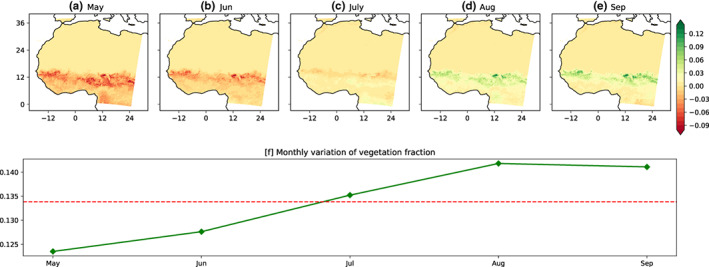
Spatial distribution of monthly difference (MV‐V minus MF‐V) in vegetation fraction (a–e) and the vegetation fraction averaged over West Africa with the red dot line showing MJJAS mean vegetation fraction (f).

Figure 2 shows the monthly differences in vegetation fraction between the MV‐V and the MF‐V from May to September. Figure 2a–e indicates less vegetation fraction in May and June compared with the MJJAS seasonal mean. Also, comparing to the seasonal mean in July, a marginal deference in vegetation fraction is noticed. Unlike the previous months where there are deficits in vegetation fraction, the months of August and September are characterized by higher vegetation fraction in the MV‐V than M‐VF. The spatial average over the West African region (Figure 2f), at the monthly scale of the vegetation fraction, from May to September, shows weakest values in May, progressively increasing through June and July, and reaches its maximum during August and September.

This seasonal trend in the vegetation fraction is consistent with the observations of Tucker et al. (1985), who noted a strong correlation between the rainfall seasonality and the dynamics of the vegetation cover, using the spectral vegetation index for different climatic zones over West Africa. Thus, the comparison of the two vegetation states reveals two main phases in vegetation variation. The first phase, from May to July, is characterized by a rapid increase in the vegetation fraction up to the value of the seasonal (MJJAS) average (0.133) as shown in Figure 2f. The second phase is characterized by an increasing vegetation fraction above the seasonal average, from July to August, where it reaches the maximum values. The most important vegetation fraction seasonal variation is recorded between 10° N and 15° N. This area represents the Sudanian Savannas and Sahelian region, a transition zone of semiarid grasslands, savannas, steppes, and thorn shrublands lying between the Sahara desert and the Sudanian Savannas (Huber & Fensholt, 2011). Previous studies focusing on the seasonal variability of the vegetation dynamics over West Africa showed that the evolution of NDVI in the Sahel region is closely related to rainfall seasonality (Anyamba & Tucker, 2005).

Moreover, there is also a good correlation between the rainfall variations and the normalized difference vegetation index (NDVI) at seasonal and interannual time scales for areas where mean annual rainfall ranges from approximately 200 to 1200 mm (Nicholson et al., 1990). This explains close links between vegetation seasonality and the displacement of the ITCZ (Anyamba et al., 2001).

### 3.2 Surface latent and sensible heat fluxes

#### 3.2.1 Impact of varying vegetation seasonal heat fluxes

**FIGURE 3:**
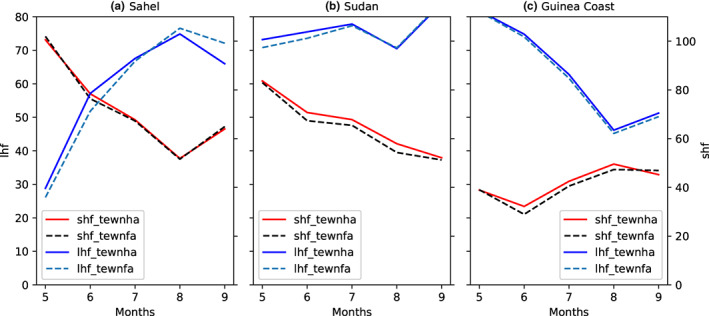
Monthly variation of the surface heat fluxes (latent and sensible heat) of fixed vegetation and variable vegetation experiment/simulation in w m^−2^ over (a) the Sahel, (b) Sudanian, and (c) Guinea coast.

**FIGURE 4:**
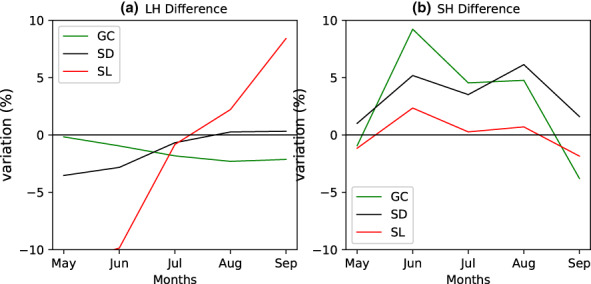
Difference (%) in latent heat flux (a) and sensible heat flux (b) over Guinea Coast (GC), Sudanian (SD), and Sahel (SL) between the simulations using MV‐V and MF‐V.

The monthly variation of latent and sensible heat fluxes over Sahel, Sudanian, and Guinea Coast is shown in Figure 3. In the Sahelian region (Figure 3a) from May, the LH, in both runs using MV‐V and MF‐V (lhf_MV‐V and lhf_MF‐V, respectively), increases gradually and reach the maximum in August. The simulated lhf_MV‐V produces higher values than the lhf_MF‐V from May to July; however, it produces slightly lower values of the maximum compared with lhf_MF‐V. Then, they decrease from August to September with higher values of LH simulated via the experiment using the MF‐V. Meanwhile, the SH in MV‐V and MF‐V simulations (shf_MV‐V and shf_MF‐V, respectively) decreases from May to July and then increases from August. Negligible differences are noticed between the shf_MV‐V and shf_MF‐V. Over Sudanian region (Figure 3b), from May to July, the LH of MV‐V and MF‐V increases gradually and reaches its maximum in July and then decreases from August to September. From May to August, the lhf_MV‐V is higher than the lhf_MF‐V. Unlike the Sahel, there is a slight difference in SH between the shf_MV‐V and shf_MF‐V. Across GC (Figure 3c), in both runs using MV‐V and MF‐V replicates similar values of LH. As noticed for the Sudanian region, there are some slight differences between the shf_MV‐V and shf_MF‐V. Varying vegetation has significantly increased the LH flux. In the Sahel, it initially increases and then suppresses the latent heat flux, while in the Sudan zone it is greater in May and June but the same as the fixed veg in July August and September. Having a lower vegetation fraction appears to increase the latent heat flux (and vice versa). This is true in both the Sahel and Sudan zones. In the Guinea coast zone and in the Aug‐July Sudan zone, the vegetation difference is minimal and so is the impact on the latent heat flux. This could be explained by some variations in surface temperature due to cloudy sky (this is the monsoon period over Sahel), unlike in Sudanian and GC where the SH is more impacted by the varying vegetation in the model. These results are in agreement with LeMone et al. (2007) who recorded maximum values of SH over sparse vegetation, which could be compared with Sudanian and Sahelian landscape and minimum values over green vegetation comparable to GC landscape; and to a lesser extent, LH maximum values occurred over green vegetation area and LH minimum over sparse vegetation. LH is more sensitive to the vegetation condition over Sahelian and Sudanian regions, thus these differences between the two simulations. The SH is sensitive to green vegetation such as vegetation over the GC. But it appears largely insensitive to the vegetation variation between simulations. Also, it is noted that during the monsoon season the response of local vegetation tends to be dampened likely due to the presence of saturated soil condition and resulting evapotranspiration amounts close to the potential regardless of the land‐cover type (Sylla et al., 2015). Overall, some variations are induced by the varying vegetation; thus, the UM is sensitive to vegetation seasonal variation. However, to show the enhancement to some extent, the performance of the model to capture the impact of vegetation variability on surface energy variation, we need some comparison with surface energy observation data. The monthly mean differences between the surface LH and SH fluxes from the simulations with MF‐V and MV‐V, over GC, SD, and SL regions, are shown in Figure 4a,b. The main differences in surface LH flux are observed over the SL region where the amplitude varies between −7.5 and 8 w m^−2^, which corresponds to variations of −10% to 10%. This area also corresponds to the region where maximum of variations is noticed in seasonal vegetation fraction and a higher vegetation fraction is associated with higher value of the surface LH flux in August–September. The lower vegetation fraction in May–June directly contributes to less evapotranspiration from the surface. This is in line with previous works which show that in wet season (monsoon period), the grass grows strongly and the increased evapotranspiration leads to a larger LH release (Moriwaki & Kanda, 2004; Steiner et al., 2009). The variations in LH over the SL region are potentially important for seasonal weather in the region because there is a strong control of African weather by the surface energy balance (Parker & Diop‐Kane, 2017) and LH fluxes represent a key exchange of moisture and energy between the land surface and the atmosphere. In contrast to SL region, the differences between the lhf_MF‐V and lhf_MF‐V relatively weak over the GC and SD regions due to the weak differences in vegetation fraction between MV‐V and MF‐V simulation.

The main variation in surface SH flux is observed over the GC and SD regions where SH increases by about 5% in June–August. SH is controlled by the difference between the aerodynamic potential temperature and air temperature as well as the surface exchange coefficient for heat. Larger values of SH correspond to lower evaporative fraction. The lowest values of SH are observed over SL region where it varies between −1 and 1 w m^−2^, a decrease of 3%. The surface SH flux is positive over GC and SD. It varies mainly from 0% to 10%. The variation in SH over the SL is relatively weak. Over SL, there is less influence of vegetation and air temperature, so the difference between MV‐V and MF‐V in terms of temperature is weak. Also, during the special observation periods (SOPs) of the AMMA Project, higher surface SH fluxes occurred before the onset and decreased to very low value during the mature monsoon period (Saïd et al., 2010). MJJAS corresponds to the period where vegetation recovers from the dry season. The two simulations show slight differences in the monthly mean of surface LH and SH fluxes. These differences are mainly observed over SL and SD regions. Over SL, MV‐V is giving the highest LH from May to July which corresponds to the period where varying vegetation fraction is weaker than the MJJAS fix vegetation fraction means. This trend is reversed in August–September when varying vegetation fraction is high. Over GC region, there is no significant variation in LH between the two simulations.

To summarize, the variation is mainly affecting the LH compared with SH and this occurs in general over SL and SD regions. Previous studies have shown that forests influence climate through the exchange of energy, water cycle, surface temperature, and SH increase with drought (Bonan, 2008). MJJAS corresponding to the period where vegetation recovers from the dry season may explain this progressive decrease in surface SH fix seasonal cycle.

#### 3.2.2 Impact of varying vegetation on diurnal heat variation

**FIGURE 5:**
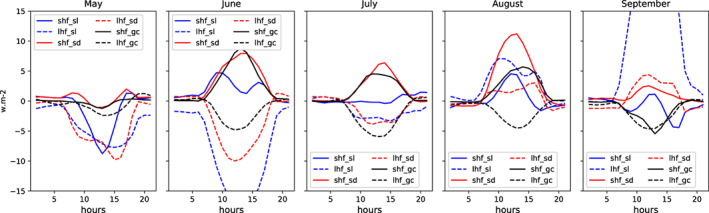
Difference between the monthly mean diurnal cycles of the MV‐V and MF‐V simulations of surface LH flux (dashed lines) in w m^−2^ and surface SH flux (Solid lines) in w m^−2^ over Sahel (SL), Sudanian Sahel (SD), and Guinea Coast (GC) regions, for each month from May to September.

The differences in LH and SH diurnal cycle fluxes (MV‐V minus MF‐V) of each month from May to September over the three climatic regions (GC, SD, and SL) are shown in Figure 5. In May, it is negative, meaning that the MV‐V gives weak SH and LH fluxes compared with MF‐V whatever climatic region is considered. In June, the LH flux difference is high with amplitudes of −5 w m^−2^, −10 w m^−2^, and beyond −15 w m^−2^, respectively, over GC, SD, and SL. However, the difference in SH is positive with 5, 8, and 10 w m^−2^ amplitude, respectively, over GC, SD, and SL. In July, the difference variation in LH flux is negative below −5 w m^−2^. The difference in SH is positive, and the amplitude has decreased. In August, both LH and SH fluxes are positive. In September, the amplitudes of LH and SH are weak in general (between −5 and 5 w m^−2^).

The GC region is characterized mainly by strong and well‐watered canopies. The daily value of surface LH flux is affected by the land–atmosphere interactions. Surface LH flux biases generally decrease (rapidly rising to its mid‐day value) until noon and then slowly increase in the afternoon until sunset (Gentine et al., 2007). Further, no significant difference is noted between vegetation fractions from MV‐V and MF‐V. This can explain why at this level, the impact of the varying vegetation on the surface LH flux is not well perceived. The amplitude of biases over this region does not vary from 1 month to another. This observation is in agreement with previous studies dealing with the land surface variability and the heat fluxes over vegetated regions. Jarlan et al. (2008) found that over forested regions, the heat signals are more stable all over the year. This dynamic of the vegetation as shown previously corresponds to the MV‐V and mainly occurs over SD and SL. This can explain the observed variation in diurnal LH flux. Over SD and SL regions, the MV‐V simulations give weak values of surface LH flux in June and July, and then, the trend is inverted in August and September where the surface LH flux biases are 5 w m^−2^ and above 10 w m^−2^, respectively. Also, over SL region the surface LH flux is higher than in SD and GC regions. So, the SL region seems to be more sensitive to surface LH flux during daytime contrary to GC regions. As we are moving into the deep monsoon from May to August, the rainfall increases therefore more moisture is accumulated in the soil during this period. Precipitation and soil moisture play a key role in LH availability and variability (Guo et al. (2011) and Song et al. (2009)). Finally, when the difference in vegetation fraction is high, the variation in LH flux is more pronounced and the daytime biases amplitude becomes more evident.

The variation of evapotranspiration can also affect the LH flux. Zheng and Eltahir (1998) have linked surface evaporation to surface water availability. Thus, the decrease in the surface water availability also reduces the surface evaporation. This trend can be explained by seasonal precipitation variability, which is accompanied by vegetation fraction growth. As the region is moving into the deep monsoon, the rainfall increases; therefore, more moisture is accumulated in the soil. Also, some of the observed bimodality could be caused by daily variation of phenology retrievals from seasonal variability (Vrieling et al., 2013). The daily cycle of the evapotranspiration seasonal variability leads to surface LH flux seasonal variability. The LH flux is low at the beginning of the season and then high when the wet period is fully set.

When it comes to the surface SH flux, over the three selected regions (GC, SD, and SL), its diurnal cycle biases increase progressively in MV‐V simulation in June, July, and August, and then it drops again in September (between −4 and 4 w m^−2^). However, in August the highest daytime variability of surface SH flux is observed over SD region 12 w m^−2^. The fact that in May surface LH and SH flux biases are all negative could be due to the simulation conditions and the spin‐up. But the spin‐up effect should be limited based on the fact that all versions of the model here are initialized with zero dust and found to be spin‐up within 5–10 days (Roberts et al., 2018). According to Cosgrove et al. (2003) without proper spin‐up, land surface simulations can be negatively impacted. Also, the observed decreases in MV‐V simulation surface SH flux could be caused by the gradual decrease in the temperature and the development of leaves during the wet season. For the surface SH flux, variations are negative in May and September and positive in June, July, and August. Contrary to the SD and SL regions, over GC region variations in surface SH flux seem to not follow any trend. This could be due to the fact that the GC region in general is permanently wet with annual rainfall amount above 1200 mm. This may have likely an impact on vegetation fraction, which seems to be stable with very less variation (Bamba et al., 2015). The surface SH flux is high during the dry period due to a dry ground surface, but decreases during the rainy period (Guo et al., 2011; Song et al., 2009).

When the surface LH flux increases during this period, the surface SH flux decreases due to increasing precipitation from monsoon. Also, the sensible heat flux is controlled in part by surface temperature. A wet surface from which water is evaporating is cooled down and so the sensible heat flux is suppressed. This is in agreement with Guo et al. (2011), who have shown that the SH flux is large during the dry ground period and decreases when the ground is wet. In contrast, LH flux increases with the wet ground but decreases with the dry ground period. Based on previous analysis, the two experiments have shown sometimes the anticorrelation relationship between surface LH and SH fluxes, which characterize these two patterns. Thus, these variations differ from one region to another. This important phenomenon may not be seen using only fixed vegetation within the model. However, the anticorrelation relationship/behavior of the LH and SH fluxes is not always fulfilled such as the case in May and August where the diurnal range of the two patterns evolves in the same direction. This could be due to the influence of other patterns such as cloudy sky, vegetation heterogeneity, and model resolution.

### 3.3 Varying vegetation sensitivity to temperature

**FIGURE 6:**
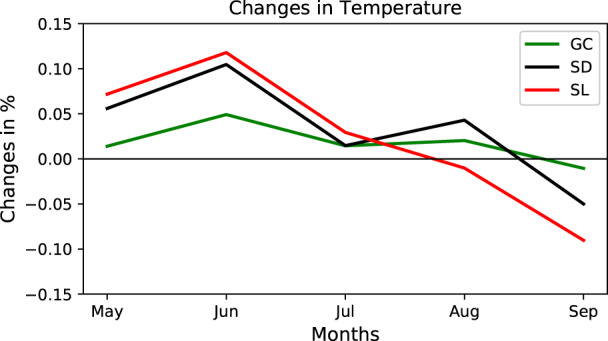
Monthly variation in temperature (in percentage) difference between MV‐V and MF‐V; and its latitudinal distribution averaged over longitudes 5W‐5E. GC, Guinea coast; SD, Sudanian; SL, Sahel.

The difference between the monthly mean air temperature at 1.5 m, between the runs using MV‐V and MF‐V, is shown in Figure 6. The highest differences are observed over SD and SL regions contrary to GC region where this variation is the weakest. Based on the monthly variation, from May to July the temperature variation seems to be regular in terms of amplitude. Over all the climatic regions, these variations in air temperature increase from May to June and then decrease from June to July and then from July to September; variations in air temperature are weak even negative from August to September. As mentioned previously in Section 3.1 in MV‐V configuration, vegetation fraction is low from May to June, which corresponds to the period where the surface temperature is the highest.

The temperature over SL and SD regions is the more sensitive (between 0.12% and −0.10% of variation, respectively) to the vegetation variation compared with GC temperatures variation (0%–0.05%). In general, the air temperature decreases from May to September.

**FIGURE 7:**
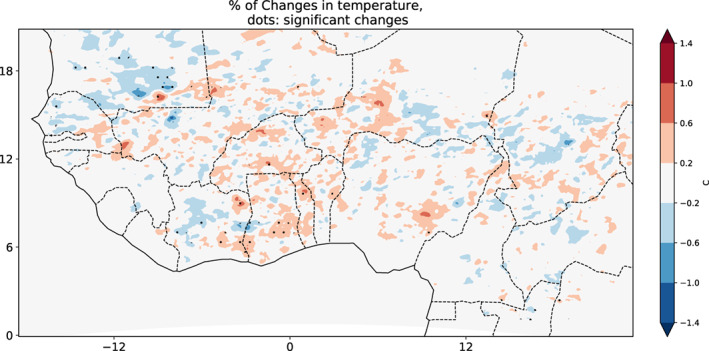
Spatial distribution of variations in surface temperature over West Africa averaged from May to September; black dots represent the areas with significant variations at *p*‐value of 10%.

The spatial distribution of temperature variations averaged over MJJAS is shown in Figure 7. Some significant variations are noted over SD precisely in Cote d'Ivoire, Ghana, and Togo. As mentioned above, varying vegetation is slightly impacting the temperature variation at a rate of 0.6 to 1°C. The air temperature decreases when vegetation fraction increases which in turn will affect the evapotranspiration. These variations are mainly negative suggesting a decrease in air temperature. Based on the above finding, we argue that running the model with varying vegetation fractions has an impact on seasonal temperature variation. The lower (higher) the vegetation fraction, the warmer (cooler) the surface temperature. Furthermore, vegetation fraction reaches its highest value in August and is followed a month later (in September) by the coldest conditions simulated. This could be due to precipitation occurrence and vegetation dynamics during this period. Vegetation fraction and precipitation may modulate the variation of surface temperature, solar radiation, and thus the energy budget. However, the feedback between vegetation and surface temperature takes some time to occur (not shown here). The surface temperature drop may not be due to the quantity of precipitation but the occurrence/continuity over time. The difference in temperature variations between the three climatic zones can be explained vegetation fraction variation in the model. The vegetation type appears to reflect soil moisture availability and that water‐use efficiency (Malo & Nicholson, 1990), indicating that these areas respond differently to multiple factors of local climate variability.

Based on the temperature seasonal cycle over West Africa which is bimodal, the monsoon period is characterized by two peaks with the MJJAS corresponding to the monsoon period. According to Afiesimama et al. (2006), the peak in temperature associated with the first mode occurs in May before the monsoon onset, and the peak of the second mode usually occurs in October as the monsoon retreats.

### 3.4 Varying vegetation sensitivity to evaporative fraction

**FIGURE 8:**
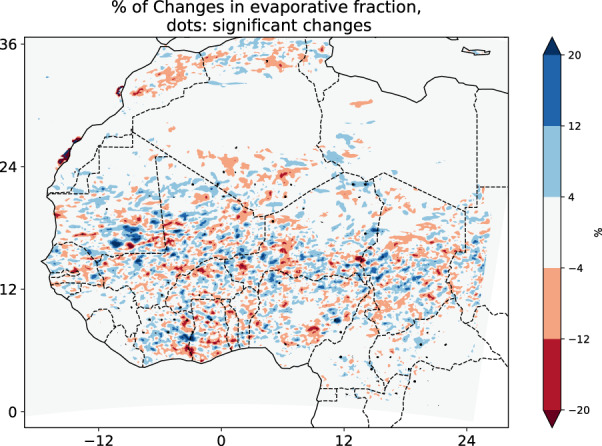
Spatial distribution of variations in evaporative fraction over West Africa averaged from May to September; black dots represent the areas with significant variations at *p*‐value of 10%.

Figure 8 shows the spatial distribution of variations in EF. Statistically significant variations in the EF are shown with dots and occur mainly over the GC region, especially in the southern part of Cote d'Ivoire, Ghana, Togo, Benin, and Nigeria. About 20% of these positive variations are located in the central part of Cote d'Ivoire up to the boundary with Ghana and vary between 0.8 and 1.2 mm day^−1^ under 12° N. Above 8° N, the EF is weak, so no significant variation is brought by the MV‐V in this region.

Below 8° N, the amount of evaporation is similar to those obtained by Opoku‐Duah et al. (2008) over the Volta basin from MODIS, AATSR, and Penman–Monteith method, respectively, 1.48, 0.47, and 2.5–2.8 mm day^−1^. Otherwise, the amplitude of evaporation fraction variation is weak in general over the three regions (0.1–0.5 mm day^−1^ over SL, 0.5 to 0.6 mm day^−1^ over SD, and 0.6 and 0.8 mm day^−1^ over GC). This is also in agreement with some previous studies which found that, during the rainy period, there was no long‐term trend (almost constant) in the evaporative fraction (Gash et al., 1997). Therefore, MV‐V has a slight impact on evaporation. As shown in Section 3.1, in terms of vegetation variation, the GC region is characterized by a marginal variation in vegetation fraction during this period.

Over SL and SD regions, the lack of significant impact of vegetation variation seems inconsistent based on the strong sensitivity of this region to rainfall variability and seasonal vegetation variation. The primary factor determining the variability of the evaporation during the wet season was the rainfall pattern. Thus, the low impact of vegetation variation in rainfall could explain the behavior of evaporation. Surface evaporation rates display distinct ranges and spatial structures, which are related in various ways to the daytime rainfall (Guichard et al., 2010).

## 4 CONCLUSION

This study assessed the sensitivities of the UM to variations in vegetation cover (fixed vegetation fraction vs. time‐varying vegetation fraction) on the simulation of the features of the West African climate, especially the surface fluxes. Convection‐permitting simulations have been performed over WAM period, which typically improves the representation of land/atmosphere coupling. The results indicate that during May–June, MV‐V has less vegetation fraction than MF‐V while during August–September, the MV‐V has high vegetation fraction than the seasonal average (MF‐V). The two simulations show some slight differences in terms of monthly variation of LH and SH. These variations are mainly observed over Sahel and Sudanian regions. The SH is higher from June to August and the LH is lower in May, June, and July when the vegetation fraction is below the seasonal mean. This trend is reversed in August–September when vegetation fraction is higher. The LH increases with vegetation fraction; this may not be seen when vegetation fraction is fixed in the climate models. The analysis suggested an impact of the varying vegetation fraction on the temperature seasonal cycle. High temperature is associated with a lower vegetation fraction, especially during May and June. This is followed by a decrease/increase in temperature as the vegetation fraction increases/decreases. It observed some differential responses between climatic variables and vegetation. They may have major implications for energy balance, vegetation response to climate anomalies, and climate variation. We conclude, therefore, that the seasonal cycle of vegetation over West Africa should be a priority for inclusion in weather prediction and climate models. Therefore, for accurate assessment of the model, long‐term data and the filtration of some phenomena should be taken into account.

## CONFLICT OF INTEREST STATEMENT

The authors declare they have no conflict of interest.

## Data Availability

The data that support the findings of this study are freely available on https://doi.org/10.5281/zenodo.7758379.

## References

[pei310107-bib-0001] Afiesimama, E. A. , Pal, J. S. , Abiodun, B. J. , Gutowski, W. J., Jr. , & Adedoyin, A. (2006). Simulation of West African monsoon using the RegCM3. Part I: Model validation and interannual variability. Theoretical and Applied Climatology, 86, 23–37. 10.1007/s00704-005-0202-8

[pei310107-bib-0002] Anyamba, A. , & Tucker, C. J. (2005). Analysis of Sahelian vegetation dynamics using NOAA‐AVHRR NDVI data from 1981–2003. Journal of Arid Environments, Special Issue on the “Greening” of the Sahel, 63, 596–614. 10.1016/j.jaridenv.2005.03.007

[pei310107-bib-0003] Anyamba, A. , Tucker, C. J. , & Eastman, J. R. (2001). NDVI anomaly patterns over Africa during the 1997/98 ENSO warm event. International Journal of Remote Sensing, 22, 1847–1860.

[pei310107-bib-0004] Bamba, A. , Diallo, I. , Touré, N. E. , Kouadio, K. , Konaré, A. , Dramé, M. S. , Diedhiou, A. , Silué, S. , Doumbia, M. , & Tall, M. (2019). Effect of the African greenbelt position on West African summer climate: A regional climate modeling study. Theoretical and Applied Climatology, 137, 309–322. 10.1007/s00704-018-2589-z

[pei310107-bib-0005] Bamba, A. , Dieppois, B. , Konaré, A. , Pellarin, T. , Balogun, A. , Dessay, N. , Kamagaté, B. , Savané, I. , & Diédhiou, A. (2015). Changes in vegetation and rainfall over West Africa during the last three decades (1981–2010). Atmospheric and Climate Sciences, 5, 367–379. 10.4236/acs.2015.54028

[pei310107-bib-0006] Best, M. J. , Pryor, M. , Clark, D. B. , Rooney, G. G. , Essery, R. L. H. , Ménard, C. B. , Edwards, J. M. , Hendry, M. A. , Porson, A. , Gedney, N. , Mercado, L. M. , Sitch, S. , Blyth, E. , Boucher, O. , Cox, P. M. , Grimmond, C. S. B. , & Harding, R. J. (2011). The Joint UK Land Environment Simulator (JULES), model description—Part 1: Energy and water fluxes. Geoscientific Model Development, 4, 677–699. 10.5194/gmd-4-677-2011

[pei310107-bib-0007] Betts, A. K. , Chen, F. , Mitchell, K. E. , & Janjić, Z. I. (1997). Assessment of the land surface and boundary layer models in two operational versions of the NCEP eta model using FIFE data. Monthly Weather Review, 125, 2896–2916. 10.1175/1520-0493(1997)125<2896:AOTLSA>2.0.CO;2

[pei310107-bib-0008] Bonan, G. B. (2008). Forests and climate change: Forcings, feedbacks, and the climate benefits of forests. Science, 320, 1444–1449. 10.1126/science.1155121 18556546

[pei310107-bib-0009] Bounoua, L. , DeFries, R. , Collatz, G. J. , Sellers, P. , & Khan, H. (2002). Effects of land cover conversion on surface climate. Climatic Change, 52, 29–64. 10.1023/A:1013051420309

[pei310107-bib-0010] Cosgrove, B. A. , Lohmann, D. , Mitchell, K. E. , Houser, P. R. , Wood, E. F. , Schaake, J. C. , Robock, A. , Sheffield, J. , Duan, Q. , Luo, L. , Higgins, R. W. , Pinker, R. T. , & Tarpley, J. D. (2003). Land surface model spin‐up behavior in the North American Land Data Assimilation System (NLDAS). Journal of Geophysical Research: Atmospheres, 108. 10.1029/2002JD003316

[pei310107-bib-0011] Gash, J. H. C. , Kabat, P. , Monteny, B. A. , Amadou, M. , Bessemoulin, P. , Billing, H. , Blyth, E. M. , deBruin, H. A. R. , Elbers, J. A. , Friborg, T. , Harrison, G. , Holwill, C. J. , Lloyd, C. R. , Lhomme, J. P. , Moncrieff, J. B. , Puech, D. , Soegaard, H. , Taupin, J. D. , Tuzet, A. , & Verhoef, A. (1997). The variability of evaporation during the HAPEX‐Sahel intensive observation period. Journal of Hydrology, 188–189, 385–399. 10.1016/S0022-1694(96)03167-8

[pei310107-bib-0012] Gentine, P. , Entekhabi, D. , Chehbouni, A. , Boulet, G. , & Duchemin, B. (2007). Analysis of evaporative fraction diurnal behaviour. Agricultural and Forest Meteorology, 143, 13–29. 10.1016/j.agrformet.2006.11.002

[pei310107-bib-0013] Guichard, F. , Asencio, N. , Peugeot, C. , Bock, O. , Redelsperger, J. L. , Cui, X. , Garvert, M. , Lamptey, B. , Orlandi, E. , Sander, J. , Fierli, F. , Gaertner, M. A. , Jones, S. C. , Lafore, J. P. , Morse, A. , Nuret, M. , Boone, A. , Balsamo, G. , de Rosnay, P. , … Bergès, J. C. (2010). An Intercomparison of simulated rainfall and Evapotranspiratio associated with a mesoscale convective system over West Africa. Weather Forecast, 25, 37–60. 10.1175/2009WAF2222250.1

[pei310107-bib-0014] Guo, D. , Yang, M. , & Wang, H. (2011). Sensible and latent heat flux response to diurnal variation in soil surface temperature and moisture under different freeze/thaw soil conditions in the seasonal frozen soil region of the central Tibetan Plateau. Environment and Earth Science, 63, 97–107. 10.1007/s12665-010-0672-6

[pei310107-bib-0015] Huber, S. , & Fensholt, R. (2011). Analysis of teleconnections between AVHRR‐based sea surface temperature and vegetation productivity in the semi‐arid Sahel. Remote Sensing of Environment, 115, 3276–3285. 10.1016/j.rse.2011.07.011

[pei310107-bib-0016] Jarlan, L. , Balsamo, G. , Lafont, S. , Beljaars, A. , Calvet, J. C. , & Mougin, E. (2008). Analysis of leaf area index in the ECMWF land surface model and impact on latent heat and carbon fluxes: Application to West Africa. Journal of Geophysical Research: Atmospheres, 113. 10.1029/2007JD009370

[pei310107-bib-0017] Kealy, J. C. , Marenco, F. , Marsham, J. H. , Garcia‐Carreras, L. , Francis, P. N. , Cooke, M. C. , & Hocking, J. (2017). Clouds over the summertime Sahara: An evaluation of met Office retrievals from Meteosat second generation using airborne remote sensing. Atmospheric Chemistry and Physics, 17, 5789–5807. 10.5194/acp-17-5789-2017

[pei310107-bib-0018] Klein, C. , Jackson, L. S. , Parker, D. J. , Marsham, J. H. , Taylor, C. M. , Rowell, D. P. , Guichard, F. , Vischel, T. , Famien, A. M. , & Diedhiou, A. (2021). Combining CMIP data with a regional convection‐permitting model and observations to project extreme rainfall under climate change. Environmental Research Letters, 16, 104023.

[pei310107-bib-0019] Kouadio, K. , Konare, A. , Diawara, A. , Dje, B. , Ajayi, V. , & Diedhiou, A. (2015). Assessment of regional climate models over Côte D'ivoire and analysis of future projections over West Africa. Atmospheric and Climate Sciences, 5, 63–81. 10.4236/acs.2015.52005

[pei310107-bib-0020] Lebel, T. , Parker, D. J. , Flamant, C. , Höller, H. , Polcher, J. , Redelsperger, J. L. , Thorncroft, C. , Bock, O. , Bourles, B. , Galle, S. , Marticorena, B. , Mougin, E. , Peugeot, C. , Cappelaere, B. , Descroix, L. , Diedhiou, A. , Gaye, A. , & Lafore, J. P. (2011). The AMMA field campaigns: Accomplishments and lessons learned. Atmospheric Science Letters, 12, 123–128.

[pei310107-bib-0021] LeMone, M. A. , Chen, F. , Alfieri, J. G. , Tewari, M. , Geerts, B. , Miao, Q. , Grossman, R. L. , & Coulter, R. L. (2007). Influence of land cover and soil moisture on the horizontal distribution of sensible and latent heat fluxes in Southeast Kansas during IHOP_2002 and CASES‐97. Journal of Hydrometeorology, 8, 68–87. 10.1175/JHM554.1

[pei310107-bib-0022] Lu, L. , Pielke, R. A. , Liston, G. E. , Parton, W. J. , Ojima, D. , & Hartman, M. (2001). Implementation of a two‐way interactive atmospheric and ecological model and its application to the Central United States. Journal of Climate, 14, 900–919. 10.1175/1520-0442(2001)014<0900:IOATWI>2.0.CO;2

[pei310107-bib-0023] Malo, A. R. , & Nicholson, S. E. (1990). A study of rainfall and vegetation dynamics in the African Sahel using normalized difference vegetation index. Journal of Arid Environments, 19, 1–24. 10.1016/S0140-1963(18)30825-5

[pei310107-bib-0024] Marsham, J. H. , Dixon, N. S. , Garcia‐Carreras, L. , Lister, G. M. S. , Parker, D. J. , Knippertz, P. , & Birch, C. E. (2013). The role of moist convection in the West African monsoon system: Insights from continental‐scale convection‐permitting simulations. Geophysical Research Letters, 40, 1843–1849. 10.1002/grl.50347

[pei310107-bib-0025] Marsham, J. H. , Parker, D. J. , Todd, M. C. , Banks, J. R. , Brindley, H. E. , Garcia‐Carreras, L. , Roberts, A. J. , & Ryder, C. L. (2016). The contrasting roles of water and dust in controlling daily variations in radiative heating of the summertime Saharan heat low. Atmospheric Chemistry and Physics, 16, 3563–3575. 10.5194/acp-16-3563-2016

[pei310107-bib-0026] Moriwaki, R. , & Kanda, M. (2004). Seasonal and diurnal fluxes of radiation, heat, water vapor, and carbon dioxide over a suburban area. Journal of Applied Meteorology, 43, 1700–1710. 10.1175/JAM2153.1

[pei310107-bib-0027] Mougin, E. , Demarez, V. , Diawara, M. , Hiernaux, P. , Soumaguel, N. , & Berg, A. (2014). Estimation of LAI, fAPAR and fCover of Sahel rangelands (Gourma, Mali). Agricultural and Forest Meteorology, 198–199, 155–167. 10.1016/j.agrformet.2014.08.006

[pei310107-bib-0028] N'Datchoh, E. T. , Diallo, I. , Konaré, A. , Silué, S. , Ogunjobi, K. O. , Diedhiou, A. , & Doumbia, M. (2018). Dust induced changes on the west African summer monsoon features. International Journal of Climatology, 38, 452–466. 10.1002/joc.5187

[pei310107-bib-0029] Nicholson, S. E. , Davenport, M. L. , & Malo, A. R. (1990). A comparison of the vegetation response to rainfall in the Sahel and East Africa, using normalized difference vegetation index from NOAA AVHRR. Climatic Change, 17, 209–241. 10.1007/BF00138369

[pei310107-bib-0030] Nogherotto, R. , Coppola, E. , Giorgi, F. , & Mariotti, L. (2013). Impact of Congo Basin deforestation on the African monsoon. Atmospheric Science Letters, 14, 45–51. 10.1002/asl2.416

[pei310107-bib-0031] Nutini, F. , Boschetti, M. , Candiani, G. , Bocchi, S. , & Brivio, P. (2014). Evaporative fraction as an indicator of moisture condition and water stress status in semi‐arid rangeland ecosystems. Remote Sensing, 6, 6300–6323. 10.3390/rs6076300

[pei310107-bib-0032] Opoku‐Duah, S. , Donoghue, D. N. M. , & Burt, T. P. (2008). Intercomparison of evapotranspiration over the Savannah Volta Basin in West Africa using remote sensing data. Sensors, 8, 2736–2761. 10.3390/s8042736 27879847 PMC3673443

[pei310107-bib-0033] Parker, D. J. , & Diop‐Kane, M. (2017). Meteorology of tropical West Africa: The forecasters' handbook. Wiley. https://www.wiley.com/enus/Meteorology+of+Tropical+West+Africa%3A+The+Forecasters%27+Handbook‐p‐9781118391303

[pei310107-bib-0034] Roberts, A. J. , Woodage, M. J. , Marsham, J. H. , Highwood, E. J. , Ryder, C. L. , McGinty, W. , Wilson, S. , & Crook, J. (2018). Can explicit convection improve modelled dust in summertime West Africa? Atmospheric Chemistry and Physics, 18, 9025–9048. 10.5194/acp-18-9025-2018

[pei310107-bib-0035] Saïd, F. , Canut, G. , Durand, P. , Lohou, F. , & Lothon, M. (2010). Seasonal evolution of boundary‐layer turbulence measured by aircraft during the AMMA 2006 special observation period. Quarterly Journal of the Royal Meteorological Society, 136, 47–65. 10.1002/qj.475

[pei310107-bib-0036] Song, Y. , Guo, W. , & Zhang, Y. (2009). Numerical study of impacts of soil moisture on the diurnal and seasonal cycles of sensible/latent heat fluxes over semi‐arid region. Advances in Atmospheric Sciences, 26, 319–326. 10.1007/s00376-009-0319-2

[pei310107-bib-0037] Steiner, A. L. , Pal, J. S. , Rauscher, S. A. , Bell, J. L. , Diffenbaugh, N. S. , Boone, A. , Sloan, L. C. , & Giorgi, F. (2009). Land surface coupling in regional climate simulations of the West African monsoon. Climate Dynamics, 33, 869–892. 10.1007/s00382-009-0543-6

[pei310107-bib-0046] Strengers, B. , Müller, C. , Schaeffer, M. , Haarsma, R. , Severijns, C. , Gerten, D. , Schaphoff, S. , van den Houdt, R. , & Oostenrijk, R. (2010). Assessing 20th century climate–Vegetation feedbacks of land‐use change and natural vegetation dynamics in a fully coupled vegetation–Climate model. International Journal of Climatology, 30, 2055–2065.

[pei310107-bib-0038] Sun, Z. , Gebremichael, M. , Ardö, J. , Nickless, A. , Caquet, B. , Merboldh, L. , & Kutschi, W. (2012). Estimation of daily evapotranspiration over Africa using MODIS/Terra and SEVIRI/MSG data. Atmospheric Research, 112, 35–44.

[pei310107-bib-0039] Sylla, M. B. , Pal, J. S. , Wang, G. L. , & Lawrence, P. J. (2015). Impact of land cover characterization on regional climate modeling over West Africa. Climate Dynamics, 46, 637–650.

[pei310107-bib-0040] Tucker, C. J. , Townshend, J. R. G. , & Goff, T. E. (1985). African land‐cover classification using satellite data. Science, 227, 369–375. 10.1126/science.227.4685.369 17815712

[pei310107-bib-0041] United Nations, Department of Economic and Social Affairs, Population Division . (2019). World population prospects 2019: Highlights (ST/ESA/SER.A/423) .

[pei310107-bib-0042] Vrieling, A. , De Leeuw, J. , & Said, M. Y. (2013). Length of growing period over Africa: Variability and trends from 30 years of NDVI time series. Remote Sensing, 5, 982–1000. 10.3390/rs5020982

[pei310107-bib-0043] Walters, D. , Baran, A. J. , Boutle, I. , Brooks, M. , Earnshaw, P. , Edwards, J. , Furtado, K. , Hill, P. , Lock, A. , Manners, J. , Morcrette, C. , Mulcahy, J. , Sanchez, C. , Smith, C. , Stratton, R. , Tennant, W. , Tomassini, L. , van Weverberg, K. , Vosper, S. , … Zerroukat, M. (2019). The met Office unified model global atmosphere 7.0/7.1 and JULES global land 7.0 configurations. Geoscientific Model Development, 12, 1909–1963. 10.5194/gmd-12-1909-2019

[pei310107-bib-0044] Weiss, J. L. , Gutzler, D. S. , Allred Coonrod, J. E. , & Dahm, C. N. (2004). Seasonal and inter‐annual relationships between vegetation and climate in Central New Mexico, USA. Journal of Arid Environments, 57, 507–534. 10.1016/S0140-1963(03)00113-7

[pei310107-bib-0045] Zheng, X. , & Eltahir, E. A. B. (1998). The role of vegetation in the dynamics of West African monsoons. Journal of Climate, 11, 2078–2096. 10.1175/1520-0442-11.8.2078

